# Isolation and partial characterization of cyclic lipopeptide antibiotics produced by *Paenibacillus ehimensis* B7

**DOI:** 10.1186/1471-2180-13-87

**Published:** 2013-04-17

**Authors:** Zhaohui Huang, Yu Hu, Linfei Shou, Mingxu Song

**Affiliations:** 1 Clinical laboratory, the Fourth Affiliated Hospital of Soochow University, 200 Huihe Road, Wuxi, 214062, China; 2 Clinical laboratory, the Fourth People’s Hospital of Wuxi, 200 Huihe Road, Wuxi, 214062, China; 3 Institute for the Control of Agro-chemicals of Zhejiang Province, 29 East Fengqi Road, Hangzhou, 310020, China

**Keywords:** *Paenibacillus ehimensis*, Lipopeptide antibiotics, Drug-resistant bacteria

## Abstract

**Background:**

The prevalence of drug-resistant bacteria has encouraged the search for novel antimicrobial compounds. Food-associated microorganisms, as a source of new antibiotics, have recently received considerable attention. The objective of this study was to find novel antimicrobial agents produced by food microorganisms.

**Results:**

A bacterial strain B7, which has potent antimicrobial activity, was isolated from a sample of dairy waste. This strain was identified as *Paenibacillus ehimensis* based on the 16S rRNA gene sequence analysis, physiological and biochemical characterization. Two active compounds (PE1 and PE2) were obtained from *P. ehimensis* B7. Mass spectrometry (MS) analysis showed that the molecular masses of PE1 and PE2 were 1,114 and 1,100 Da, respectively. The tandem MS and amino acid analysis indicated that PE1 and PE2 were analogs of polypeptin, and PE2 was characterized as a new member of this family. Both compounds were active against all tested bacterial pathogens, including methicillin resistant *Staphylococcus aureus*, *Escherichia coli*, and pan-drug resistant *Pseudomonas aeruginosa* clinical isolate. Time-kill assays demonstrated that at 4 × MIC (minimum inhibitory concentration), PE1 and PE2 rapidly reduced the number of viable cells by at least 3-orders of magnitude, indicating that they were bactericidal antibiotics.

**Conclusions:**

In the present work, two cationic lipopeptide antibiotics (PE1 and PE2) were isolated from *P. ehimensis* B7 and characterized. These two peptides showed broad antimicrobial activity against all tested human pathogens and are worthy of further study.

## Background

Clinical infection due to drug-resistant bacteria is a serious challenge to patient safety [1,2]. In the United States, methicillin-resistant *Staphylococcus aureus* (MRSA) is estimated to cause ~19,000 deaths per year [3]. MRSA is also a considerable threat in China, where the resistance ratio among hospital-acquired infections reaches almost 90% [4,5]. Apart from MRSA, several multidrug-resistant (MDR) and pan-drug-resistant (PDR) Gram-negative bacteria, including *Pseudomonas aeruginosa*, *Escherichia coli*, *Acinetobacter baumannii*, and *Klebsiella pneumoniae*, are emerging as additional major threat to public health [6,7]. Unfortunately, few novel drugs have been developed specifically for MDR/PDR Gram-negative bacteria in recent years [8-10]. The development of new antimicrobial agents cannot keep up with the evolution of bacterial resistance. Thus, more efforts should be placed on discovering and developing new antimicrobial agents.

As a source of new antibiotics, food-associated microorganisms have recently received increased attention. The well-known active compounds produced by these strains are peptide antibiotics, such as lantibiotics and lipopeptides [11-13]. Many of them are potentially useful in medical and food applications due to their low intestinal toxicity. To obtain antimicrobial agents that are novel safe and potent, a lot of food bacteria were isolated and screened for their antimicrobial activity. In this work, strain B7, a new bacterial isolate from a sample of dairy waste, was found to produce antibiotics against both Gram-positive and Gram-negative human pathogens. Based on the 16S rRNA gene sequence analysis as well as physiological and biochemical characterization, strain B7 was identified as *Paenibacillus ehimensis*. After isolation and purification of the fermentation products, the chemical structure and biological characteristics of the active compounds produced by *P. ehimensis* B7 were determined.

## Methods

### Strains and culture conditions

Samples of dairy waste were collected from a local dairy industry in Wuxi. The dairy waste samples were suspended in 0.1% sterile peptone water and antibiotic producing strains were isolated using a competitive inhibition method as previously described [14]. Nutrition broth was used for routine culture. The active compounds were produced in synthetic Katznelson and Lochhead (KL) medium, which had the following composition (in g/L): glucose, 5; (NH_4_)_2_SO_4_, 1.5; MgSO_4_^.^7H_2_O, 0.2; NaCl, 0.1; CaC1_2_, 0.1; FeSO_4_^.^7H_2_O, 0.01; ZnSO_4_, 0.01; MnSO_4_^.^H_2_O, 0.0075; and KH_2_PO_4_ 2.7. The medium was autoclaved and brought to a pH of 7.2. *Staphylococcus epidermis* CMCC 26069 was purchased from the National Center for Medical Culture Collections. *S. aureus* ATCC 43300, *S. aureus* ATCC 25923, *E. coli* ATCC 35218, and *P. aeruginosa* ATCC 27853 were purchased from the American Type Culture Collection (ATCC). Clinical isolates (*P. aeruginosa* 5215 and *E. coli* 5539) were isolated from patients at the Fourth People’s Hospital of Wuxi, Wuxi, China. The tested strains that were used to determine the sensitivity to the active compounds were routinely grown at 37°C on a nutrient agar or in a nutrient broth. For long-term storage, all of the strains were stored in 20% (v/v) glycerol at −80°C. This study was approved by the Ethics Committee of the Fourth People’s Hospital of Wuxi.

### Strain identification

The morphology of strain B7 was examined by light microscopy after Gram-staining and spore staining. The physiological and biochemical characteristics of the isolate was assessed according to previously described methods [15]. Motility was determined using sulfide-indole-motility medium. Fatty acid methyl esters were extracted and analyzed by the Sherlock Microbial Identification system (MIDI, Newark, DE) according to the manufacturer’s instructions. All assays were performed in triplicate. The 16S rRNA gene of strain B7 was amplified by PCR with the universal primers 27F and 1541R and sequenced [16]. Phylogenetic trees were constructed using the neighbor-joining and maximum-parsimony algorithm within MEGA4 [17]. The DNA-DNA hybridization between B7 and *Paenibacillus ehimensis* IFO 15659^T^ was performed using the thermal denaturation method [14].

### Production and purification of active compounds

Strain B7 maintained on nutrient agar slants was inoculated into 50 mL of nutrient broth and cultivated at 30°C for 24 h. The seed culture of strain B7 was transferred to a 2L Erlenmeyer flask that contained 500 mL of the KL medium. The culture was incubated on a rotary shaker (200 rpm) at 30°C for 3 d. After centrifugation at 4500 g for 30 min at 4°C, the cell-free supernatant was loaded onto a column packed with Amberlite XAD-16 resin (Sigma, St. Louis, MO). The column was washed with distilled water prior to elution with stepwise gradients of aqueous methanol (30, 60, and 100%, v/v). Each fraction was concentrated and assessed for activity using the paper disc method. The active fraction was evaporated and dried before being redissolved in acetonitrile. The concentrated solution was then applied to a C_18_ SPE column (Hardwee, Germany). The column was washed with five bed volumes of distilled water, followed by five bed volumes of an acetonitrile/water mixture (20:80, v/v). The fraction that contained the active compounds was eluted from the column by washing with three bed volumes of an acetonitrile/water mixture (68:32, v/v). Further purification was performed using a preparative HPLC system (Dalian Elite, Dalian, China) that was equipped with an YMC-pack DOS-A C18 (5 μm, 250 × 20 mm) column. The mobile phase consisted of Milli-Q water that contained 0.02% trifluoroacetic acid and acetonitrile. A linear gradient of 15% to 55% acetonitrile (40 min) was used for elution at a flow rate of 10 mL/min. UV detection was performed at a wavelength of 210 nm. Fractions from multiple runs were collected and combined for the subsequent antimicrobial activity assays. The active fractions were passed through the HPLC column two consecutive times.

### Amino acid analysis

Approximately 300 μg of the purified compound in 0.4 ml of 6 M HCl with 0.1% phenol was hydrolyzed at 110°C for 16 h. Amino acid analyses was performed using ion-exchange chromatography with a Hitachi L-8900 amino acid analyzer (Tokyo, Japan) according to the method described by Qian et al. [18]. The absolute configuration of amino acids was determined using 1-fluoro-2,4-dinitrophenyl-5-D-leucinamide (D-FDLA) and 1-fluoro-2,4-1-fluoro-2,4-dinitrophenyl-5-L-leuci-namide (L-FDLA) as derivatizing agents. FDLA derivative analysis was performed as previously described [19].

### Mass spectrometry analysis

Electrospray ionization (ESI) mass spectra were acquired in positive ion mode on a Thermo Finnigan LCQ mass spectrometer (Thermo Electron Corporation, San Jose, CA, USA). The ESI-mass spectrometry (MS) conditions included a capillary voltage of 40 V, a source voltage of 4.5 kV, and a capillary temperature of 300°C. To obtain the amino acid sequences, collision induced dissociation (CID) was applied to the purified lipopeptide antibiotics.

### Antibacterial activity assay

During fermentation and purification, antimicrobial activity was determined using the paper disc method [14]. The minimum inhibitory concentrations (MICs) of the purified antibiotics were determined using a microbroth dilution method according to the National Committee for Clinical Laboratory Standards (2009). The final concentrations of the antibiotics in the medium ranged from 1 to 64 μg/mL. MICs were measured after incubation at 37°C for 20 h. To determine the effect of divalent cations on the mode action of purified compounds, 10 mM CaCl_2_ or MgCl_2_ was added to the test medium.

### Time-kill assays

To further evaluate the antimicrobial characteristics of the purified compounds, time-kill experiments were performed as previously described [18]. The active compound was added to a logarithmic-phase broth culture of approximately 10^6^ cfu/mL to yield concentrations of 0 and 4× MIC. The cultures were incubated with shaking (120 rpm) at 37°C for 24 h. Surviving bacteria were determined after 0, 1, 3, 6, and 24 h of incubation by subculturing 100 μL serial dilutions of samples in 0.9% sodium chloride on MH agar plates. A bactericidal effect was defined as a ≥ 3 log10 cfu/mL decrease compared with the initial inoculum.

### Cytotoxicity assay

Cytotoxicity analysis was performed on the HEK293 human embryonic kidney cell line using the Cell Counting Kit-8 (CCK-8; Dojindo, Tokyo, Japan). The HEK293T cells were seeded into 96-well plates at 1 × 10^4^ cells/well. After incubation for 24 h at 37°C in a humidified atmosphere, the medium was replaced with fresh medium that contained active compound (1 μg/mL to 128 μg/mL, in 2-fold increments). Three replicate wells were set for each treatment. After incubation for another 48 h, cell growth was assayed with CCK-8. The relative absorbance was recorded at 450 nm.

### Nucleotide accession number

The nucleotide sequence of 16S rRNA gene of strain B7 has been deposited in GenBank under the accession number JX282195.

## Results

### Identification of strain B7

The bacteria strain B7 that is active against MRSA ATCC 43300 and *P. aeruginosa* ATCC 27853 was selected for further investigation. Morphologically, strain B7 was characterized to be a rod-shaped, spore-forming, motile, Gram-positive bacterium. Aerobic growth of B7 occurred at a temperature between 20 and 50°C and a pH between 6 and 8. Optimal conditions included a temperature of 30°C and a pH of 7. The bacteria strain B7 was negative for urease and positive for catalase, oxidase, methyl red test, and nitrate reduction. Starch, chitin, and gelatin were hydrolyzed by strain B7. Acid was produced from D-mannitol, D-gentiobiose, D-xylose, D-Mannose, L-arabinose, mannitol, and glucose. The G + C content of the strain DNA was 54.2%. The major fatty acid of strain B7 was anteiso-C_15:0_, making up to 50.12% of the total fatty acids, a characteristic of the genus *Paenibacillus*. The B7 isolate and *P. ehimensis* IFO 15659^T^ showed identical 16S rRNA gene sequences [20], which suggests that they are members of the same species. This inference was further confirmed by the DNA-DNA hybridization results. The DNA-DNA re-association between strain B7 and *P. ehimensis* IFO 15659^T^ was 96.3%. All of these characteristics supported the identification of the isolate as a member of *P. ehimensis.* Thus, strain B7 was named *P. ehimensis* B7.

### Purification of antibiotics produced by P. ehimensis B7

*P. ehimensis* B7 grew well and produced active compounds in the KL medium. Bioactivity was detectable approximately 20 h after inoculation and reached a maximum level at 96 h. The cultures were separated into supernatant and cell pellets by centrifugation. Before purification, the stability of the antibiotics that were present in the culture supernatant was investigated according to a previously described method [15]. The active compounds were stable at a pH of 2.0 to 8.0, and their antimicrobial activities were also not affected by heat treatment at 40 or 80°C for 1 h.

The antibiotics were easily absorbed from the culture supernatant by Amberlite XAD-16 resin. The resin was washed with distilled water and then eluted with stepwise gradients of aqueous methanol. One fraction that was eluted with 100% methanol exhibited the most significant antimicrobial activity. This fraction was extracted with a SPE cartridge and further separated by HPLC. Two active compounds that were eluted at retention times of 28.2 and 26.4 min were obtained and named PE1 and PE2, respectively. The final yield was approximately 17.6 mg/L for PE1 and 12.3 mg/L for PE2.

### Structure analysis

ESI-MS analysis indicated that PE1 had a molecular mass of 1114 Da, and PE2 had a molecular weight of 1,100 Da. The two molecular masses differed from each other by 14 Da, suggesting that they were homologues. Amino acid analysis demonstrated that these two compounds had the same amino acid composition, and both of them contained L- 2,4-diaminobutyric acid (L-Dab), L-leucine (L-Leu), L-isoleucine (L-Ile), L-threonine (L-Thr), D-Phenylalanine (D-Phe), and D-valine (D-Val), with molar ratios of 3:2:1:1:1:1, which further confirmed that they were structural close-related peptide antibiotics.

**Figure 1:**
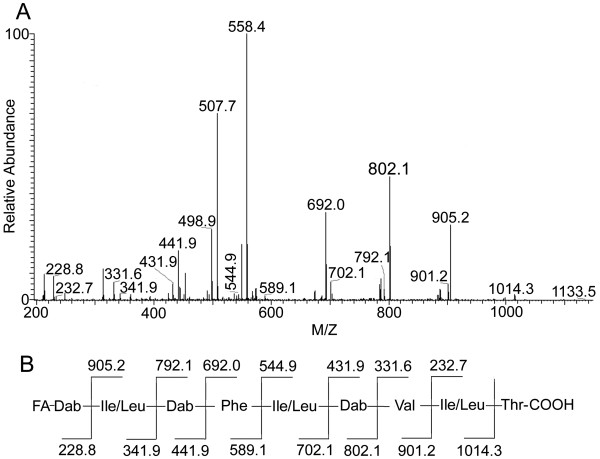
**MS/MS spectrum of PE1 and its proposed amino acid sequence. (A)** MS/MS spectrum of the doubly charged precursor ion at m/z 567.4 of the hydrolyzed PE1 of 1,133 Da. **(B)** Proposed amino acid sequence of PE1.

After alkaline hydrolysis (1 M KOH, 8 h, room temperature), the purified compounds with protonated molecular masses of 1,115 and 1,101 Da, yielded two new products, with [M + H]^+^ ions of 1,133 and 1,119 Da, respectively. The 18 Da increase in mass was attributed to the hydrolysis of a lactone. This result indicated that the two compounds were cyclic lipopeptide antibiotics. The MS/MS spectrum of the doubly charged precursor ion of the hydrolyzed compound at m/z 567.4 with a mass of 1133 Da was shown in Figure 1. Successive fragmentations from the two termini of the ring-opened lipopeptide resulted in b-type ions at m/z 1014.3, 901.2, 802.1, 702.1, 589.1, 441.9, 341.9, and 228.8, along with corresponding y-type ions detected at m/z 905.2, 792.1, 692.0, 544.9, 431.9, 331.6, and 232.7. These fragment ions allowed for the assignment of the following sequence: Ile/Leu-Dab-Phe- Leu/Ile-Dab-Val-Leu/Ile-Thr-OH. The b-type ions at m/z 228.8 corresponded to fatty acid (FA)-Dab, which indicated that the fatty acyl moiety has the elemental composition of C_7_H_12_O_2_.

**Figure 2:**
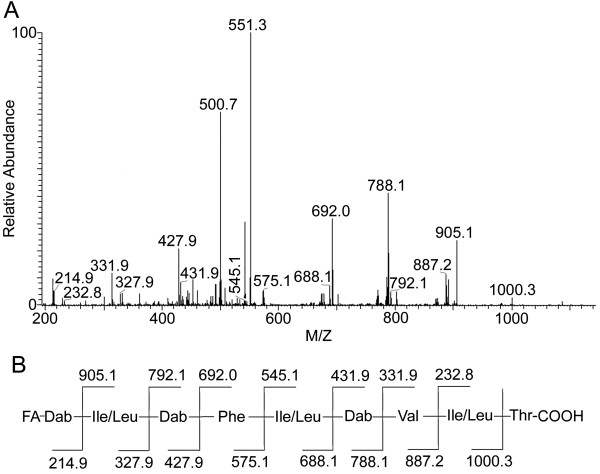
**MS/MS spectrum of PE2 and its proposed amino acid sequence. (A)** MS/MS spectrum of the doubly charged precursor ion at m/z 560.3 of the hydrolyzed PE2 of 1,119 Da. **(B)** Proposed amino acid sequence of PE2.

The ring-opened PE2 with a mass [M + H]^+^ of 1,119 Da was also analyzed by CID. The tandem mass spectrum of this derivative was shown in Figure 2. All of the b-type ions that were generated from this doubly charged precursor ion [M + 2H]^2+^ at m/z 560.3 were 14 Da less than those generated from the precursor ion [M + 2H]^2+^ at m/z 567.4. However, the two y-type ion series for the two compounds were almost the same in mass, which indicated that the two compounds had identical amino acid sequences but different fatty acid chains. Similar to PE1, PE2 also produced a fragment ion at m/z 905.1, which corresponded to the loss of 214 Da from the [M + H]^+^ ion. Examination of the neutral fragment that was lost suggested that it contains a Dab residue and a fatty acyl moiety (C_6_H_10_O_2_). These results further confirmed that the two compounds were different in their fatty acyl moieties.

**Figure 3:**
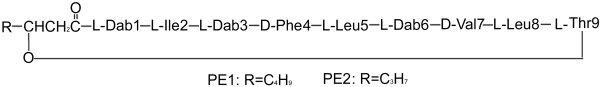
**Proposed structures of PE1 and PE2 produced by *Paenibacillus ehimensis *B7.**

Apart from in the C-terminal amino acid (Thr), no hydroxyl group was found in the peptide moieties of *P. ehimensis* lipopeptides studied here. Thus, a lactone linkage was only formed between the carboxyl group of the C-terminal and the hydroxyl group of fatty acid moieties. The proposed structures for PE1 and PE2 are showed in Figure 3.

### Antimicrobial activities of the purified compounds

The antimicrobial activities of the purified compounds PE1 and PE2 were measured using micro dilution methods. Table 1 showed that PE1 and PE2 both had a similar level of strong activity against all of the tested Gram-positive and Gram-negative pathogens as well as *Candida albicans*. The MICs of the compounds were lower than those of commercial polymyxin B in the cases of Gram-positive bacteria. In the case of *E. coli* ATCC 35318, *E. coli* 5539, and *P. aeruginosa* ATCC 27853, the MICs of PE1 and PE2 were higher than that of polymyxin B. Interestingly, *P. aeruginosa* 5215, a pan-drug resistant clinical isolate, was highly sensitive to PE1 and PE2, with MICs of 2 μg/mL that was slightly lower than that of polymyxin B.

**Table 1:** **The minimum inhibitory concentrations (MICs) of lipopeptide antibiotics (PE1 and PE2) produced by *Paenibacillus ehimensis *B7**

**Indicator strain**	**MIC (μg/mL)**		
	**PE1**	**PE2**	**polymyxin B**
*Staphylococcus epidermidis* CMCC 26069	1	1	4
*Staphylococcus aureus* ATCC 25923	8	8	64
*Staphylococcus aureus* ATCC 43300	4	4	32
*Escherichia coli* ATCC 35318	8	8	2
*Escherichia coli* 5539	4	4	1
*Pseudomonas aeruginosa* ATCC 27853	8	4	2
*Pseudomonas aeruginosa* 5215	2	2	4
*Candida albicans* ATCC 10231	8	8	64

### Time-kill assays

**Figure 4:**
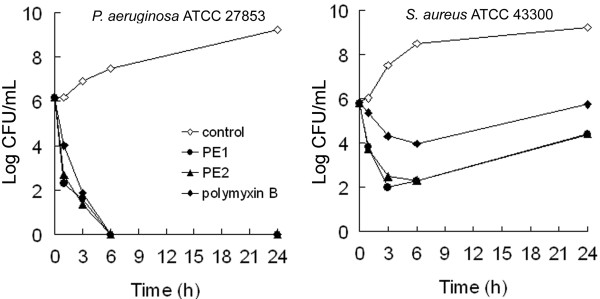
**Growth curves of *Pseudomonas aeruginosa *ATCC 27853 and *Staphylococcus aureus *ATCC 43300 treated with 4 × MIC peptide antibiotics.** The curves are viable cell concentrations plotted against time. In two panels, non-antibiotic control, open diamond; 4 × MIC PE1, filled circle; 4 × MIC PE2, filled triangle; 4 × MIC polymyxin B, filled diamond. For the two strains in the present study, time-kill assays were independently performed 3 times and similar results were obtained. Mean values of the triplicate cfu/mL measurements from a single experiment are plotted.

To further evaluate the growth inhibition effect of newly isolated antibiotics, killing experiments of PE1 and PE2 against *S. aureus* ATCC 43300 and *P. aeruginosa* ATCC 27853 were performed. The time-kill curves of PE1 against both strains were similar to PE2 (Figure 4). In the case of *P. aeruginosa* ATCC 27853, all of the tested antibiotics at 4 × MIC rapidly reduced the number of viable cells of this strain by at least 3 orders of magnitude over the first 3 h of exposure, and no bacteria could be detected after a 24 h incubation. In the case of *S. aureus* ATCC 43300, the number of viable cells counted also dramatically decreased within a period of 3 h following the addition of these two compounds, although substantial re-growth occurred after 24 h. Thus, PE1 and PE2 were determined to be bactericidal at high concentrations, which is consistent with the characteristics of other cationic cyclic lipopeptides [21,22].

### Effect of divalent cations on antibacterial activity

To determine the effect of divalent cations on the antibacterial activity of the lipopeptides that are produced by *P. ehimensis* B7, the MICs of PE1 against *S. aureus* ATCC 43300 and *P. aeruginosa* ATCC 27853 were determined in MH medium with 10 mM Ca^2+^ or Mg^2+^. In normal medium, the MICs of PE1 for *S. aureus* ATCC 43300 and *P. aeruginosa* ATCC 27853 were 4 and 8 μg/mL, respectively. However, the MICs of PE1 for *S. aureus* ATCC 43300 and *P. aeruginosa* ATCC 27853 increased to 8 and >64 μg/mL, respectively, when 10 mM CaCl_2_ was added to the test medium. Similar results were obtained when Mg^2+^ was added instead of Ca^2+^ (Table 2).

**Table 2:** **Effect of divalent cations on antibacterial activity of lipopeptide antibiotics (PE1 and PE2) produced by *Paenibacillus ehimensis *B7**

**Antibiotic**	**MIC (μg/mL)**	
	***P. aeruginosa *ATCC 27853**	***S. aureus *ATCC 43300**
PE1	8	4
PE1 + 10 mM CaCl_2_	>64	8
PE1 + 10 mM MgCl_2_	>64	8

### Cytotoxicity

**Figure 5:**
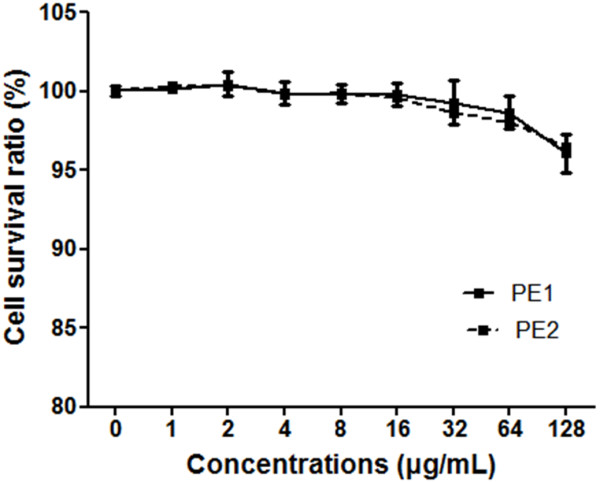
**Cytotoxicity of PE1 and PE2 to mammalian cells.** Cytotoxicity of PE1 and PE2 to HEK293T was measured with the CCK-8 assay. The concentrations of PE1 and PE2 ranged from 0 to 128 μg/mL. The positive control was 0.1% Triton X-100.

The cytotoxicity of the purified compounds (PE1 and PE2) against mammalian cells was tested by the CCK-8 assay. PE1 and PE2 showed little cytotoxicity against HEK293T cells (treatment time, 24 h) at all of concentrations that were tested (1 μg/mL to 128 μg/mL) (Figure 5).

## Discussion

In the present study, B7, a new bacterial strain with potent antimicrobial activity was isolated from a dairy waste sample, and identified as *P. ehimensis*. Phylogenetic analysis based on 16S rRNA gene indicated that the isolate was closely related to *P. elgii*, *P. koreensis*, and *P. tianmuensis* (data not shown). This group of bacteria produces diverse antimicrobial agents, including lipopeptides [15,22,23], lantibiotics [24] and macrolide [14]. Interestingly, most extensively described lipopeptide antibiotics from this group of bacteria contain a high percentage of both Dab and a C6-C7 N-terminal fatty acyl chain [15,22].

The active compounds (PE1 and PE2) that are produced by *P. ehimensis* B7 were structurally similar to the lipopeptide polypeptins (A and B) that were previously isolated from *Bacillus circulum*[25]. Polypeptin is a group of polypeptide antibiotics composed of a cyclic nonapeptide moiety and a fatty acid side chain. To date, five polypeptin-type antibiotics, including polypeptin A, polypeptin B, permetin A, BMY-28160, and pelgipeptin D, have been extensively described [25-28]. Polypeptins A and B, which have the same molecular formula, have identical amino acid moieties but vary in the structure of fatty acids. BMY-28160 and permetin A only differ from each other at position 2 in peptide moieties (i.e., L-Val is in BMY-28160, and L-Ile in permetin A). Pelgipeptin D and permetin A only differ from each other in the fatty acid moiety, while permetin A differs from polypeptin A only in the amino acid at position 9 (i.e., L-Ser is present in permetin A, and L-Thr in polypeptin A). Polypeptin-type antibiotics were known to have a broad spectrum of antimicrobial activity against many Gram-positive and Gram-negative bacteria [25]. The molecular mass of PE1 was identical to that of polypeptin A and B, and the amino acid sequences and antimicrobial spectra were extremely similar, suggesting PE1 and polypeptin (A or B) are most likely the same compound. However, PE2 was characterized as a new analog of polypeptin because PE2 and polypeptin contained identical peptide moieties, but differed from each other by a molecular mass of 14 Da in fatty acid chains. Although Ile and Leu cannot be distinguished by tandem mass spectrometry, there are two reasons for us to assign Ile at position 2, and Leu at positions 5 and 8 in the PE peptide antibiotics (Figure 4). Firstly, the amino acid compositions of PE and polypeptin are identical, including the molar ratios of amino acids and their absolute configurations. Secondly, positions 5 and 8 of the peptide moieties in all of the extensively described members of polypeptin are Leu, whereas Ile or Val is present in their peptide moieties at position 2 [15,25].

The active modes of cationic lipopeptides generally involve the interaction of positive charged residues with bacterial cell wall, which is normally stabilized by divalent cations (Ca^2+^ and Mg^2+^) [8,29,30]. This is consistent with our results that the addition of 10 mM Ca^2+^ or Mg^2+^ significantly reduced the susceptibility of Gram-negative and Gram-positive bacteria to lipopeptides from *P. ehimensis.* In addition to positive residues, the fatty acyl chain and amphipathic structure also contribute to the antimicrobial activity of cationic peptides [12,31]. Although polypeptin and polymyxin are structurally related cyclic lipopeptides with several basic amino acids, their antimicrobial potencies and spectra are significantly different. Polypeptin has a broad-spectrum activity against Gram-positive and Gram-negative bacteria, whereas polymyxin is potently active mainly against Gram-negative bacteria. The selectivity of lipopeptide antibiotics may be attributable to their differential binding affinities to the external and/or cytoplasmic membrane of Gram-negative and Gram-positive bacteria. Understanding the action mode of polypeptin may provide some useful clues toward developing novel lipopeptide antibiotics.

## Conclusion

In conclusion, two active compounds (PE1 and PE2) were obtained from the newly isolated strain *P. ehimensis*. Structural analysis indicated that they were analogs of polypeptin, and PE2 was characterized as a novel analog of polypeptin. These two compounds showed potent activity against Gram-positive and Gram-negative bacterial pathogens, including MRSA and pan-drug resistant *P. aeruginosa*. Although the present results provide some valuable information about the cyclic lipopeptide antibiotics that are produced by *Paenibacillus ehimensis*, further studies are needed to determine their potential clinical utility.

## Competing interests

The authors declare to have no competing interests.

## Authors’ contributions

ZH was responsible for designing the study and writing the manuscript. ZH and YH performed the strain selection and identification experiments. LS and MS carried out the purification and identification of lipopeptide antibiotics. All authors read and approved the final manuscript.
